# Supplementary material for: Transmission, Pathological and Clinical Manifestations of Highly Pathogenic Avian Influenza A Virus in Mammals with Emphasis on H5N1 Clade 2.3.4.4b
Source: Viruses. 2025 Nov 26;17(12):1548. doi: 10.3390/v17121548 (PMC12737373; doi:10.3390/v17121548)
Supplement: Supplementary file 1 [file viruses-17-01548-s001.zip › viruses-3997584-supplementary.pdf]

Tables S1, S2 and S3. Transmission, pathological and clinical manifestations of highly pathogenic avian influenza A virus in mammals with emphasis on H5N1 clade 2.3.4.4b

Table S1. Summary of studies describing domesticated mammals.

Study and species	Clinical signs	Respiratory lesions	CNS lesions	Gastrointestinal lesions	Lesions in other organs	Virus localization (RT-qPCR)	Virus localization (IHC)	Transmission route
Burrough et al., 2024<br><br>Dairy cow (*Bos taurus*), United States of America. (n=6)	Non- specific illness, reduced feed intake, drop in milk production and dehydration. Good BCs (n=6/6).	NA	NA	Macro: Small abomasal ulcers and erosions of the intestines.<br>Micro: Abomasitis (n= 4/6), probably age-related.	Macro:Mammary gland lesions (n= 6).<br>Micro: Multifocal suppurative mastitis (n=2/6), hepatitis (n=3/6).	Low Ct values for virus RNA in milk and mammary gland homogenates (Ct = 12.3- 16.9) compared to other tissues. (n=2/6) OBS only received mammary tissue from 2 cows.	IAV NP antigen present in mammary gland with nuclear and cytoplasmic staining of alveolar epithelial cells and in multifocal germinal centers of lymphoid tissue.	Feed contaminated with feces from wild birds + potentially horizontal transmission.

IAV: Influenza A Virus. NA: not available. Macro: macroscopic pathological lesions. Micro: microscopic pathological lesions. PCR: polymerase chain reaction. IHC: immunohistochemistry. Ct: Cycle threshold. NP: Nucleoprotein.

Table S1 continued

Study and species	Clinical signs	Respiratory lesions	CNS lesions	Lesions in other organs	Virus localization (RT- qPCR)	Virus localization (IHC)	Transmission route	Study and species
Burrough et al., 2024<br><br>Cat (*Felis catus*), United States of America. (n=2)	Found dead. Adequate BCs (2/2)	Micro: Multifocal Interstitial pneumonia (n=2/2).	Macro: Meningeal hemorrhages in cerebrum (n=2/2) Micro: Multifocal meningoencephalitis, vasculitis and neuronal necrosis (n=2/2).	NA	Macro: Hemorrhages in the subcutaneous tissue (n=2/2) Micro: Multifocal myocarditis (n=2/2), vacuolar hepatopathy (n=2/2) and multifocal chorioretinitis (n=1/2).	Low Ct values for virus RNA in brain (Ct = 9.9 – 13.5) and lung (Ct = 17.4 – 24.4). tissue.	IAV NP antigen present in brain, lung, heart (n=2/2) and retina (n=1/2).	Feeding on unpasteurized colostrum from infected cows.
Kang et al., 2024<br><br>Cat (*Felis catus*), South Korea.<br><br>(n=9)	Respiratory and neurological signs (n= 5/9).<br><br>Deceased (n=4/9).<br><br>BCS: normal (n= 4/4).	Macro: Congestion and edema in the lungs (n=3/4). Micro: Interstitial pneumonia	Micro: Multifocal meningoencephalitis, neuronal necrosis and gliosis (n= ¼).	Macro: Bloody diarrhea in small intestine (n=1/4). Micro: Enteritis.	Macro: Jaundice in eyes (n= ¾). Pericardial effusion and hemorrhage (n= 4/4). Splenomegaly (n= ¾). Necrotic	Virus RNA is present in a large range of tissues. *(Specific Ct values in a supplementary table)*	IAV NP antigen present in alveolar macrophages and bronchial epithelial cells in the lung (n= 4/4), in neurons and glial cells in CNS (n= ¼) and intestine	"Improperly sterilized raw food made from domestic duck meat."

IAV: Influenza A Virus. NA: not available. Macro: macroscopic pathological lesions. Micro: microscopic pathological lesions. PCR: polymerase chain reaction. IHC: immunohistochemistry. Ct: Cycle threshold. NP: Nucleoprotein.

Table S2. Summary of studies describing terrestrial mammals.

Study and species	Clinical signs	Respiratory lesions	CNS lesions	Lesions in other organs	Virus localization (RT-qPCR)	Virus localization (IHC)	Transmission route
Elsmo et al., 2023<br><br>Wild terrestrial mammals in the United states (n= 67). Found alive (n=58). Gross postmortem observations (n= 58). Histopathological examination (n= 55).	Neurological signs (n= 56/58), lethargy (n= 28/58), fever (n= 7/58), diarrhea (n= 2/58), unconsciousness (n= 2/58), recumbence (n= 1/58), paralysis (n= 1/58), vomiting (n= 1/58) and dyspnea (n=4/58). Good nutritional condition (n =39/58).	Macro: Lung lesions (n= 49/58) including congestion (n= 42), edema (n= 22), failure to collapse (n= 18), hemorrhage (n= 18) and pleural effusion (n= 6). Micro: Multifocal Interstitial pneumonia (n= 47/55). Few animals had bronchopneumonia.	Macro: hemorrhage (n = 11/58) and congestion (n =7/58). Micro: Multifocal regions of malaci and inflammation, neural necrosis often associated with satellitosis or karyorrhectic debris. Meningoencephalitis (n=51/55).	Macro Nematode parasitism (n= 15/58), congestion, hemorrhage in GI tract and loose feces. Pallor (n= 8/58), Congestion (n= 7/58),enlargement (n=6/58) and hemorrhage (n=1/58) in the liver. Congestion (n=7/58) and cortical hemorrhage (n= 1/58) in the kidney. Pericardial effusion (n= 3/58), petechiae (n= 2/58) and myocardial pallor (n= 2/58). Micro: Multifocal myocardial necrosis and fibrosis (n= 29/55). Mineralization of affected Cardiomyocytes. Random foci of acute liquefactive to coagulative hepatic necrosis (n= 22/55). Lymphoid depletion (n = 28/55).	Low Ct values for virus RNA in the brain compared to other tissues (n= 21/35).	Tested on 29 animals. IAV NP antigen present in brain (17/22), lung, heart and liver tissue	Spillover from infected wild birds.

IAV: Influenza A Virus. NA: not available. Macro: macroscopic pathological lesions. Micro: microscopic pathological lesions. PCR: polymerase chain reaction. IHC: immunohistochemistry. Ct: Cycle threshold. NP: Nucleoprotein.

Table S2 continued.

**Study and species**	**Clinical signs**	**Respiratory lesions**	**CNS lesions**	**Lesions in other organs**	**Virus localization (RT- qPCR)**	**Virus localization (IHC)**	**Transmission route**
Elsmo et al., 2023<br><br>Red fox (Vulpes vulpes), United States of America (n= 50	Dyspnea (n= 1).	NA	NA	Macro: Gastric contents that included feathers (n= 3).<br>Micro: Multifocal pancreatitis and pancreatic necrosis (n= 1).	NA	IAV NP antigen is present in brain tissue (n= 13/17), in the cerebral cortex (n= 13/13), thalamus (n= 6/13), hippocampus (n= 3/13) and brain stem (n= 1/13).<br>Mild staining of epithelial cells and interstitial macrophages in lung tissue (n=2/15). Present in Cardiac myofibers and interstitial macrophages surrounding foci of necrosis (n= 3/5). In the liver (n= 1/3).	NA
Elsmo et al., 2023<br><br>Striped skunks (Mephitis mephi tis), United States of America. (n= 6)	Dyspnea (n= 2).	NA	Macro:<br>No brain lesions (n= 2).<br><br>Micro: Brain lesions (n= ⅗).	Micro: Lymphoid necrosis in the spleen (n= 5), lymph nodes (n= 4) and peyer's patches (n= 1).	Virus RNA detected in brain tissue (n = ⅗), lung (n= 3/3) and liver (n= 2/2).	IAV NP antigen is present in the lung with mild staining of epithelial cells and interstitial macrophages (n= 2/2). In hepatocytes surrounding necrotic foci (n= 5/5).	Spillover from infected wild birds.

IAV: Influenza A Virus. NA: not available. Macro: macroscopic pathological lesions. Micro: microscopic pathological lesions. PCR: polymerase chain reaction. IHC: immunohistochemistry. Ct: Cycle threshold. NP: Nucleoprotein.

Table S2 continued.

**Study and species**	**Clinical signs**	**Respiratory lesions**	**CNS lesions**	**Lesions in other organs**	**Virus localization (RT- qPCR)**	**Virus localization (IHC)**	**Transmission route**
Elsmo et al., 2023<br><br>Raccoons (Procyon lotor), United States of America. (n= 4)	NA	Lung worm parasitism (n=3/4)	Micro: Brain lesions observed (n= 4/4).	Micro: Lesions in the lymphoid system in general (n = 2/4). Heart lesions (n= 1/4). Liver lesions (n=1/4).	Virus RNA detected in brain tissue (n= 1/1).	IAV NP antigen present in lung with mild staining of epithelial cells and interstitial macrophages in lung tissue (n= 1/1). In cardiac myofibers and interstitial macrophages surrounding foci of necrosis (n= 1/1). In brain tissue (n= 1/1) and in lymphoid tissue (n= 1/1).	Spillover from infected wild birds.
Elsmo et al., 2023<br><br>Bobcats (Lynx rufus), United States of America. (n= 2)	Dyspnea (n=1)	Lung worm parasitism (n=1)	NA	NA	NA	NA	Spillover from infected wild birds.

IAV: Influenza A Virus. NA: not available. Macro: macroscopic pathological lesions. Micro: microscopic pathological lesions. PCR: polymerase chain reaction. IHC: immunohistochemistry. Ct: Cycle threshold. NP: Nucleoprotein.

Table S2 continued.

**Study and species**	**Clinical signs**	**Respiratory lesions**	**CNS lesions**	**Lesions in other organs**	**Virus localization (RT- qPCR)**	**Virus localization (IHC)**	**Transmission route**
Elsmo et al., 2023<br><br>Virginia opossums (*Didelphis virginiana*), United States of America. (n= 2)	NA	NA	Micro: Meningitis (n= 1/2).	Liver lesions observed (n= 1/2).	Virus RNA detected in brain tissue (n= 2/2).	IAV NP antigen present in lung within pneumocytes and to a lesser extent alveolar macrophages and bronchial and bronchiolar epithelium (n= 1/2) and in brain tissue (n= 2/2).	Spillover from infected wild birds.
Elsmo et al., 2023<br><br>Coyote (*Canis latrans*), United States of America. (n= 1)	NA	NA	NA	NA	NA	NA	Spillover from infected wild birds.

IAV: Influenza A Virus. NA: not available. Macro: macroscopic pathological lesions. Micro: microscopic pathological lesions. PCR: polymerase chain reaction. IHC: immunohistochemistry. Ct: Cycle threshold. NP: Nucleoprotein.

Table S2 continued.

Study and species	Clinical signs	Respiratory lesions	CNS lesions	Lesions in other organs	Virus localization (RT- qPCR)	Virus localization (IHC)	Transmission route
Elsmo et al., 2023<br><br>Gray fox (*Urocyon cinereoargenteus*), United States of America. (n= 1)	NA	NA	No brain lesions.	Severe hemorrhage in all body cavities. No heart lesions.	NA	NA	Spillover from infected wild birds.
Jakobek et al., 2023<br><br>Black bears (*Ursus americanus*), North america. (n= 2, one cub and 1 adult female).	Active and vocal (cub). Neurological signs include wandering between vehicles, falling into a river, circling, recumbency and convulsing (adult).	NA	Macro: Congestion of cerebrum (cub). Micro: Multifocal meningitis, neural necrosis associated with satellitosis and glial nodules and axonal degeneration (adult + cub but less intense). And fibrinoid vasculitis (cub).	Macro: Thin and limited fat stores (cub). Mesenteric lymphadenomegaly (cub). Micro: Small necrotic foci of the liver (cub).	Virus RNA detected in Brain tissue and tracheo- rectal swabs.	IAV NP antigen present in the brain (adult + cub) and liver cells (cub).	Spillover from infected wild birds.

IAV: Influenza A Virus. NA: not available. Macro: macroscopic pathological lesions. Micro: microscopic pathological lesions. PCR: polymerase chain reaction. IHC: immunohistochemistry. Ct: Cycle threshold. NP: Nucleoprotein.

Table S2 continued.

Study and species	Clinical signs	Respiratory lesions	CNS lesions	Lesions in other organs	Virus localization (RT-qPCR)	Virus localization (IHC)	Transmission route
Falchieri et al., 2024<br><br>Bush dogs (*Speothos venaticus*), United Kingdom. (n=10)	Hindlimb ataxia, forelimb hypermetria, depression and polyuria (n= 10/10).	Macro: Intracardiac euthanasia made gross assessment of the lungs challenging (n= 6/10). Multifocal hemorrhage (n= .). Micro: Debris in bronchial lumen and necrosis of bronchial. Alveoli is filled with inflammatory cells and fibrin. Involvement of alveolar septae including vasculitis (n= 6/10).	Macro: Meninges expanded by multifocal inflammation (n= 4/4). Micro: Meningeal Infiltration of inflammatory cells (n= 4/4). Necrosis and inflammation of cerebrum, brainstem and cerebellum (n= 4/4). Multifocal vasculitis in meninges and neuropil (n= 4/4).	Macro: Congestion and hemorrhage of intestines (n= 4/10). Hepatomegaly (n= 8/10) and Multifocal discolouration of liver lobes (n= 7/10). Ascites (n= 7/10). Bilateral adrenomegaly (n= 6/10). Splenomegaly n= 3/10). Micro: Multifocal vascular changes of arterioles and veins (n= 10/10). Multifocal necrosis and inflammation of the liver (n= 10/10). Multifocal necrosis and inflammation of adrenal glands (n= 3/?). Multifocal to diffuse lymphadenitis (n= 3/3). Multifocal splenitis (n= 4/10). Anterior uveitis, optic neuritis and retinitis (n= ?). Mild necrosis and degeneration of heart (n= 3/10) and pancreas (n= 2/10). Multifocal to diffuse fibrinous peritonitis of many abdominal organs.	Virus RNA detected in a broad range of tissues.	IAV NP antigen present in macrophages (in the periphery of necrosis and inflammation of affected organs) and in vessel walls. In the liver (n= 6/10). In ependymal cells lining the ventricles and in meninges of cerebrum (n= 5/10), cerebellum (n= 3/10) and in brainstem (n= 1/10). In bronchial epithelium (n= 6/10). In the adrenal cortex (n= 2/10). In the eye (n= 3/10). Strong labelling in the smooth muscle layers of the intestine (n= 2/10).	Ingestion of infected meat.

IAV: Influenza A Virus. NA: not available. Macro: macroscopic pathological lesions. Micro: microscopic pathological lesions. PCR: polymerase chain reaction. IHC: immunohistochemistry. Ct: Cycle threshold. NP: Nucleoprotein.

Table S2 continued.

Study and species	Clinical signs	Respiratory lesions	CNS lesions	Lesions in other organs	Virus localization (RT- qPCR)	Virus localization (IHC)	Transmission route
Lagan et al., 2023<br><br>Red fox (*Vulpes vulpes*), Ireland. (n= 2).	Found dead.	NA	Micro: non-suppurativ meningoencephalitis with associated lym lymphocytic vasculitis, perivascular lymphocytic cuffing and endothelial necrosis (n= 2/2).	NA	Highest detection of virus RNA in the brain (Ct = 18.73). Present in respiratory, intestine and viscera tissue. And in oropharyngeal swabs.	IAV NP antigen present in large numbers in the brain/cerebrum.	Spillover from infected wild birds.
Bordes et al., 2023<br><br>Red fox (*Vulpes vulpes*), Netherlands. (n= 3)	Neurological signs including blindness, head shaking, falling over and opisthotonus (n= 1). Convulsions (n= 1). Lethargy (n=2). All were adult males with moderate to poor BCS.	Macro: Failure to collapse with marbled red aspect (n= 3/3). Micro: The nasal conchae and trachea displayed suppurative inflammation with the presence of parasitic structures (*Capillaria spp*.) Micro: purulent rhinitis (1/2)	Micro: Nonsuppurative encephalitis with perivascular cuffing (n=3/3).	Micro: Subacute lymphoplasmacytic myocarditis with myocardial degeneration and myocardial necrosis (n= ⅓).	Virus RNA is detected in large numbers in the brain (Ct = 26.23, 22.19 and 17.77). No virus RNA detected in rectal swabs and low numbers in throat swabs (Ct= 30.31, 23.33 and 26.81). For one fox were comparable virus RNA loads detected in the brain samples and throat swab (n= 1).	IAV NP antigen present in large numbers in the brain (n= 3/3). IAV-positive olfactory epithelial cells with necrosis of these cells (n= 1/3) and in cardiomyocytes (n= 1/3).	Feeding on/spillover from infected birds.

IAV: Influenza A Virus. NA: not available. Macro: macroscopic pathological lesions. Micro: microscopic pathological lesions. PCR: polymerase chain reaction. IHC: immunohistochemistry. Ct: Cycle threshold. NP: Nucleoprotein. BCS: body condition score.

Table S2 continued.

Study and species	Clinical signs	Respiratory lesions	CNS lesions	Lesions in other organs	Virus localization (RT- qPCR)	Virus localization (IHC)	Transmission route
Rodriguez et al., 2024<br><br>South American Coatis (*Nasua nasua*), South America. (n= 23).<br>**Group 1: f**emales and an intact male living in an outdoor enclosure (n= 5 survives), while **group 2:** composed of castrated males housed in a roofed and enclosed area.	Depression and lethargy followed by sudden death (n= 18).	NA	NA	NA	Virus RNA detected in oropharyngeal (Ct= 24.1) (n= 1), rectal (Ct= 24.3) (n= 1) and encephalon (Ct = 18.3 and 20.1) (n= 2) samples from 3 dead animals (n= 3/3).	NA	Direct or indirectly from wild birds in an ecological park.

IAV: Influenza A Virus. NA: not available. Macro: macroscopic pathological lesions. Micro: microscopic pathological lesions. PCR: polymerase chain reaction. IHC: immunohistochemistry. Ct: Cycle threshold. NP: Nucleoprotein.

Table S2 continued.

**Study and species**	**Clinical signs**	**Respiratory lesions**	**CNS lesions**	**Lesions in other organs**	**Virus localization (RT- qPCR)**	**Virus localization (IHC)**	**Transmission route**
Liang et al., 2023<br><br>Red Fox, (*Vulpes Vulpes*), Denmark. (n= 4). 1 adult and 3 cubs.	The adult fox was in poor body condition, while the body conditions of the fox cubs were within normal range.	Macro: pulmonary edema and consolidation(n= 4/4). Pronounced emphysema in the cranial parts (n=3/4 cubs).<br>Micro: Fibrinous tonecrotizing pneumonia(n= 1/4, adult), varying degrees of fibrinous to interstitial pneumonia (n= 3/4, cubs).	NA	Macro: Liver enlarged with congestion (n= 4/4) and hepatic steatosis (n= ¾ cubs).	Virus RNA detected in oropharyngeal (Ct= 24.1) (n= 1), rectal (Ct= 24.3) (n= 1) and encephalon (Ct = 18.3 and 20.1) (n= 2) samples from 3 dead red foxes.	NA	Direct or indirectly from wild birds in an ecological park.

IAV: Influenza A Virus. NA: not available. Macro: macroscopic pathological lesions. Micro: microscopic pathological lesions. PCR: polymerase chain reaction. IHC: immunohistochemistry. Ct: Cycle threshold. NP: Nucleoprotein.

Table S2 continued.

**Study and species**	**Clinical signs**	**Respiratory lesions**	**CNS lesions**	**Lesions in other organs**	**Virus localization (RT- qPCR)**	**Virus localization (IHC)**	**Transmission route**
Bessière et al., 2023<br><br>Tibetan black bear (*Ursus thibetanus*), France (n=1).	Dyspnea, hyperthermia, lateral decubitus (lethargy), diarrhea. Biochemical profile revealed acute renal failure (leukopenia and hypercreatinemia)	Macro: "severe congestion of lungs and necrotic tracheal mucosa". Micro: "pulmonary edema and congestion; moderate suppurative tracheitis with submucosal vascular thrombosis".	NA	Macro: "Severe congestion of intestines. hemorrhagic lesions on the epicardium and liver". Micro: "Acute, marked multifocal to coalescing fibrino-necrotizing lymphadenitis and splenitis with vasculitis and hemorrhages. Severe, multifocal necro-suppurative hepatitis. Marked acute hemorrhages, on the subepicardium and renal interstitium".	No RT-qPCR but in situ hybridization (detecting M gene): Trachea (submucosal and rare endothelial cells), lung, heart, spleen, stomach (subserosal connective tissue), intestine (myenteric plexus nerve trunk and connective tissue - rare intraluminal debris).	"IAV NP detected in lymph node, lung (luminal debris and interlobular mesenchymal cells), myenteric plexus nerve trunk and connective tissue, kidney."	Not elucidated but most likely spillover from infected birds (direct or indirect contact).

IAV: Influenza A Virus. NA: not available. Macro: macroscopic pathological lesions. Micro: microscopic pathological lesions. PCR: polymerase chain reaction. IHC: immunohistochemistry. Ct: Cycle threshold. NP: Nucleoprotein.

Table S2 continued.

Study and species	Clinical signs	Respiratory lesions	CNS lesions	Lesions in other organs	Virus localization (RT- qPCR)	Virus localization (IHC)	Transmission route
Hiono et al,. 2023<br><br>Tanuki (Nyctereutes procyonoides albus).	"Severely emaciated and moderately dehydrated. Showed depression and blindness and eye discharge."	Macro: Anthracosis of lungs. Micro: "Infiltration of neutrophils, lymphocytes and macrophages in trachea. Infiltration of lymphocytes around bronchi and brinchioles."	Macro: no lesions observed. Micro: "Loss of Purkinje cells and proliferation of Bergmann glia."	"Nematodes in stomach and larva migrans.<br>bilateral conjunctivitis with clouded lenses. Focal 0.8 x 0.4 yellow-white focus in the liver. Discolored kidneys.<br>Micro: Severe fatty degeneration in tubule epithelium of the kidney with dilated lumen and flattened epithelial lining. Multifocal infiltration of lymphocytes and neutrophils in renal cortex.<br>Degenerated and necrotic neutrophils with bacterial masses in the palpebral fissue. Infiltration of neutrophils and lymphocytes in the lamina propria of the bulbar<br>and palpebral conjunctiva of the upper eyelid.<br>Other: Larva migrans (nematodes observed in liver, lungs and spleen."	NA	NA	NA

IAV: Influenza A Virus. NA: not available. Macro: macroscopic pathological lesions. Micro: microscopic pathological lesions. PCR: polymerase chain reaction. IHC: immunohistochemistry. Ct: Cycle threshold. NP: Nucleoprotein.

Table S2 continued.

Study and species	Clinical signs	Respiratory lesions	CNS lesions	Lesions in other organs	Virus localization (RT- qPCR)	Virus localization (IHC)	Transmission route
Hiono et al,. 2023<br><br>An ezo red fox (*Vulpes vulpes schrencki*), Japan.	Deceased. Emaciated and severely dehydrated.	"Pale or reddish-brown lungs with diffuse and mild edema. Micro: Fibrin deposition in alveoli. Infiltration of neutrophils, lymphocytes and macrophages."	Macro: No lesions observed. Micro: "mild-to-moderate localized meningoencephalitis with infiltration of neutrophils in the olfactory bulb, corpus striatum, occipital lobe, cerebellum, and mesencephalon. Focal necrosis in occipital lobe and mesencephalon."	Macro: "Multiple yellow-white foci with 1 mm in size in liver and with a sizes of 5 x 3 mm in pancreas". Micro: "Multiple focal necrosis in hepatocytes with surrounding neutrophil infiltration. Vacuolar degeneration of hepatocytes with bile plug formation and bile pigmentation with similar lesions in kidney proximal and distal tubules expect for neutrophil infiltration. Focal necrotic lesions in pancreas with scattered hemorrhage and infiltration of neutrophils and macrophages".	NA	NA	Ingestion of infected birds.

IAV: Influenza A Virus. NA: not available. Macro: macroscopic pathological lesions. Micro: microscopic pathological lesions. PCR: polymerase chain reaction. IHC: immunohistochemistry. Ct: Cycle threshold. NP: Nucleoprotein.

Table S3. Summary of studies describing marine mammals.

Study and species	Clinical signs	Respiratory lesions	CNS lesions	Lesions in other organs	Virus localization (RT- qPCR)	Virus localization (IHC)	Transmission route
Pardo-Roa et al., 2025<br><br>Common dolphin (*Delphinus delphis*), Peru (n= 2)	Deceased	NA	NA	NA	NA	NA	Spillover from infected wild birds.
Pardo-Roa et al., 2025<br><br>Marine otter (*Lontra felina*), Peru (n= 2)	Deceased	NA	NA	NA	NA	NA	Spillover from infected wild birds.
Pardo-Roa et al., 2025<br><br>Burmeister's porpoises (*Phocoena spinipinnis*), Peru (n= 2)	Deceased	NA	NA	NA	NA	NA	Spillover from infected wild birds.

IAV: Influenza A Virus. NA: not available. Macro: macroscopic pathological lesions. Micro: microscopic pathological lesions. PCR: polymerase chain reaction. IHC: immunohistochemistry. Ct: Cycle threshold. NP: Nucleoprotein.

Table S3 continued.

**Study and species**	**Clinical signs**	**Respiratory lesions**	**CNS lesions**	**Lesions in other organs**	**Virus localization (RT- qPCR)**	**Virus localization (IHC)**	**Transmission route**
Pardo-Roa et al., 2025<br><br>Chilean dolphins (*Cephalorhy nchus eutropia*), Peru (n= 2)	Deceased	NA	NA	NA	NA	NA	Spillover from infected wild birds.
García- García et al., 2017<br><br>Harbor porpoise (*Phocoena phocoena*), Sweden (n=1)	Swimming in circles, unable to right itself and drowned shortly after discovery	Macro: Pulmonary edema (drowning)<br><br>Micro: Mononuclear septal thickening. Increased number of alveolar macrophages.	Micro: Meningoencep halitis + neuronal necrosis, gliosis, perivascular cuffing. Vasculitis.	NA	Virus RNA detected in brain (Ct = 20.57), lungs (Ct= 30.73), kidney (Ct= 31.37), liver (Ct= 32.75) and Spleen (Ct= 33.43).	IAV NP antigen present in the brain in multifocal areas in nuclei and cytoplasm of neurons, glial cells, epithelial cells of choroid plexus. In macrophages in alveoli of lung tissue.	Spillover from infected wild birds.

IAV: Influenza A Virus. NA: not available. Macro: macroscopic pathological lesions. Micro: microscopic pathological lesions. PCR: polymerase chain reaction. IHC: immunohistochemistry. Ct: Cycle threshold. NP: Nucleoprotein.

Table S3 continued.

Study and species	Clinical signs	Respiratory lesions	CNS lesions	Lesions in other organs	Virus localization (RT- qPCR)	Virus localization (IHC)	Transmission route
Ulloa et al., 2023<br><br>South American sea lion (*Otaria flavescens*),<br>Peru (n= 21),<br>necropsy of (n= 2).	Tremors, stumbling, stiffness of the neck, nystagmus, weakness and paralysis, dyspnea (n= 1/2).<br><br>Severe weight loss, muscle wasting, emaciation, weakness (n= 1/2).	Macro: Hyperemic lungs, atelectasis, emphysematous areas (n= 1/2).<br><br>Micro: Multifocal pneumonia (n= 2/2).	Macro: Hyperemic vessels (n= 1/2)<br><br>Micro: Multifocal non-suppurative meningoencep halitis, encephalitis, gliosis, neuronophagia, neuronal and glial necrosis (n= 2/2).	Macro: Congestion of mesenteric vessels (n= 1/2). Congestion of liver, enlarged mesenteric lymph nodes (n= 1/2).<br><br>Micro: Focal myocarditis and pancreatitis (n= 1/2).	Virus RNA detected in brain (Ct= 18.4), lungs (Ct= 26.8), liver (Ct= 30.6), kidney (Ct= 27.3), kidney (Ct= 25.2) and heart (Ct= 31.4). With negative swab samples. (case 1).<br><br>Virus RNA detected in lung (Ct= 21), pancreas (Ct= 31.9), liver (Ct= 23.8), urinary bladder (Ct= 33.7), heart (Ct=34.8).<br><br>With positive swab samples: Rectal (Ct= 32.7) and tracheal (Ct= 31.9)<br>(case 2)	IAV antigen found in brain tissue in neurons, glial cells and inflammatory cells (n= 2) and lung tissue (n= 1).	Spillover from infected wild birds.<br><br>Suspect direct transmission between sea lions.

IAV: Influenza A Virus. NA: not available. Macro: macroscopic pathological lesions. Micro: microscopic pathological lesions. PCR: polymerase chain reaction. IHC: immunohistochemistry. Ct: Cycle threshold. NP: Nucleoprotein.

Table S3 continued.

**Study and species**	**Clinical signs**	**Respiratory lesions**	**CNS lesions**	**Lesions in other organs**	**Virus localization (RT- qPCR)**	**Virus localization (IHC)**	**Transmission route**
Lair et al., 2024<br><br>Harbor Seal (*Phoca vitulina*), Quebec, Canada. (n=14)	Lethargy and neurologic signs. Observations of weak and dyspneic harbor seals reported	Macro: Red-tinged foam in the tracheal lumen, and pulmonary congestion (n= 14/14).<br>Micro: Pulmonary inflammatory changes (n=11/15)<br>Acute multifocal fibrinosuppurative alveolitis (n=9/10).<br>Interstitial pneumonia (n=8/15).<br>Alveolar emphysema (n=4/15), alveolar damage (n=6/15).	Micro: Multifocal to diffuse meningoencephalitis with neutrophilic and lymphocytic infiltrates.<br>Neuronal necrosis, satellitosis and gliosis (n= 14/14).	Macro: Lymphadenomegaly.<br><br>Micro: Multifocal adrenocortical necrosis (n=6/10).<br>Acute multifocal necrotizing thymitis (n=2/4), lymphadenitis (n=5/14) and splenitis (n=3/14) along with lymphoid depletion.<br>Mild multifocal membranous glomerulonephritis (n=6/15).<br>Necrotizing fibrinous hepatitis (n=4/14).<br>Mild multifocal myocarditis (n=1/14).	NA	IAV NP antigens found in neurons, lungs, glomerulus, spleen, pancreas, liver, skeletal muscle, lymph nodes, trachea and adrenal glands. (n=12/13)	Spillover from infected wild birds. Ingestion of birds or feces/feathers from birds.

IAV: Influenza A Virus. NA: not available. Macro: macroscopic pathological lesions. Micro: microscopic pathological lesions. PCR: polymerase chain reaction. IHC: immunohistochemistry. Ct: Cycle threshold. NP: Nucleoprotein.

Table S3 continued.

**Study and species**	**Clinical signs**	**Respiratory lesions**	**CNS lesions**	**Lesions in other organs**	**Virus localization (RT- qPCR)**	**Virus localization (IHC)**	**Transmission route**
Lair et al., 2024<br><br>Gray seal (*Halichoerus grypus*), Quebec, Canada (n = 1)	Deceased	Macro: Red-tinged foam in the tracheal lumen, and pulmonary congestion.<br><br>Micro: acute fibrinosuppurative alveolitis. Interstitial pneumonia. Alveolar emphysema, alveolar damage, necrotic type1 pneumocytes.	Micro: Meningoencephalitis. Neuronal necrosis, satellitosis, gliosis.	Macro: Lymphadenomegaly.<br><br>Micro: multifocal necrotic foci of the adrenal cortex. Acute necrotizing, thymitis, lymphadenitis, splenitis.<br><br>Glomerulonephritis and necrotizing fibrinous hepatitis. Myocarditis.	NA	IAV NP antigens found in: Neurons, lungs, glomerulus, spleen, pancreas, liver, skeletal muscle, lymph nodes, trachea and adrenal glands. Detections were often associated with foci of necrosis.	Spillover from infected wild birds. Ingestion of birds or feces/feathers from birds.
Mirolo et al., 2023<br><br>Grey seal (*Halichoerus grypus*), Netherlands (n=1)	Deceased	Macro: Dark-red coloration of lungs.<br><br>Micro: Mild focal pneumonia.	Micro: Acute multifocal lympho-histiocytic and necrotising encephalitis, gliosis and neuronal necrosis.	NA	Virus RNA detected in the brain (Ct= 16.60).	IAV NP antigens detected only in brain tissue.	Spillover from infected wild birds.

IAV: Influenza A Virus. NA: not available. Macro: macroscopic pathological lesions. Micro: microscopic pathological lesions. PCR: polymerase chain reaction. IHC: immunohistochemistry. Ct: Cycle threshold. NP: Nucleoprotein.

Table S3 continued.

Study and species	Clinical signs	Respiratory lesions	CNS lesions	Lesions in other organs	Virus localization (RT- qPCR)	Virus localization (IHC)	Transmission route
Mirolo et al., 2023<br><br>Grey seal (*Halichoerus grypus*), Germany (n=1)	Deceased	Macro: Lungs failed to collapse. Diffuse firmnes, red parenchyma.<br><br>Micro: Multifocal non- suppurativ interstitial pneumonia and lympho-histiocytic vasculitis.	Micro: Multifocal to coalescing lympho-histiocytic meningoencep halitis, lympho-histiocytic to necrotising vasculitis and single cell necrosis in neuroparenchy ma.	Macro: Enteritis. Hyperplastic lymph nodes, thymic atrophy, perisplenitis, perihepatitis.<br>Micro: Depletion of lymphocytic organs and vasculitis in several organs.	Virus RNA detected in lung and brain tissue.	IAV NP antigen detected only in brain tissue in neuron and glial cells.	Infected after admission to a rehabilitation center by gulls.
Uhart et al., 2024<br><br>Elephant seals (*Mimrounga leonina*), South America (n = >17.000 pups and unknown amount of adults)	NA	NA	NA	NA	NA	NA	First introduction to the population is unclear.<br><br>Mammal-to-mammal transmission

IAV: Influenza A Virus. NA: not available. Macro: macroscopic pathological lesions. Micro: microscopic pathological lesions. PCR: polymerase chain reaction. IHC: immunohistochemistry. Ct: Cycle threshold. NP: Nucleoprotein.

Table S3 continued.

**Study and species**	**Clinical signs**	**Respiratory lesions**	**CNS lesions**	**Lesions in other organs**	**Virus localization (RT- qPCR)**	**Virus localization (IHC)**	**Transmission route**
Uhart et al., 2024<br><br>Elephant seal pups (*Mimrounga leonina*), South America<br>(n = 4)	Lethargic, difficulties rolling or galumphing. Labored breathing, nasal discharge, repetitive head or flipper movements and tremors.	Macro: Foam or mucoid nasal discharge and foam in trachea (could be due to drowning).	NA<br>(Due to biosecurity!)	NA<br>(Due to biosecurity!)	NA<br>(Due to biosecurity!)	NA<br>(Due to biosecurity!)	Mammal-to-mammal transmission.<br>As the pups are toothless and nurtured solely on milk.<br>Minimal contact with birds.<br>Transplacental?
Uhart et al., 2024<br><br>Elephant seal sub adult male (*Mimrounga leonina*), South America<br>(n = 1)	Tremors, labored breathing, yellowish and blood-stained nasal discharge, hyperthermia.	NA<br>(Due to biosecurity!)	NA<br>(Due to biosecurity!)	NA<br>(Due to biosecurity!)	NA<br>(Due to biosecurity!)	NA<br>(Due to biosecurity!)	NA<br>(Due to biosecurity!)

IAV: Influenza A Virus. NA: not available. Macro: macroscopic pathological lesions. Micro: microscopic pathological lesions. PCR: polymerase chain reaction. IHC: immunohistochemistry. Ct: Cycle threshold. NP: Nucleoprotein.

Table S3 continued.

Study and species	Clinical signs	Respiratory lesions	CNS lesions	Lesions in other organs	Virus localization (RT- qPCR)	Virus localization (IHC)	Transmission route
Fiorito et al., 2025<br><br>Southern elephant seal (*Mirounga leonina*), Argentina (n = 1)<br><br>NEC 94	Deceased. Good BCS.	Macro: Congestion, atelectatic and with abundant blood and reddish foam. High viscosity mucus in larynx and serosanguinous fluid in the thorax.<br><br>Micro: Granulomatous bronchopneumonia (probably due to bacterial infection).	Macro: Meningeal congestion and multifocal hemorrhages.<br><br>Micro: Lymphoneutrophilic meningitis and multifocal areas of encephalitis. Neuronal and glial cell degeneration and necrosis. Neurophagia, glial cells aggregates and perivascular cuffing.	Macro: hemopericardium.<br><br>Micro: Multifocal myocardial necrosis, inflammation and calcification.	Virus RNA detected in cerebral cortex (Ct=28.4, n=1/3), and heart (Ct=36.4, n=1/3).	IAV NP antigen present in necrotic and inflammatory lesions in the cytoplasm and nuclei of degenerated neurons and glial cells.<br>Present in cytoplasm and nucleus of cardiomyocytes<br><br>No IAV NP antigen present in lung tissue.	NA
Uhart et al., 2024<br><br>Elephant seal sub adult male (*Mimrounga leonina*), South America (n = 1)	Tremors, labored breathing, yellowish and blood-stained nasal discharge, hyperthermia.	NA (Due to biosecurity!)	NA (Due to biosecurity!)	NA (Due to biosecurity!)	NA (Due to biosecurity!)	NA (Due to biosecurity!)	NA (Due to biosecurity!)

IAV: Influenza A Virus. NA: not available. Macro: macroscopic pathological lesions. Micro: microscopic pathological lesions. PCR: polymerase chain reaction. IHC: immunohistochemistry. Ct: Cycle threshold. NP: Nucleoprotein

Table S3 continued.

Study and species	Clinical signs	Respiratory lesions	CNS lesions	Lesions in other organs	Virus localization (RT- qPCR)	Virus localization (IHC)	Transmission route
Fiorito et al., 2025<br><br>South American sea lion, Argentina (*Otaria flavescens*)<br><br>(n = 3)<br>NEC 83,<br>NEC 85 and<br>NEC 86.	Difficulty swimming (n=1/3), dyspnea, nasal and oral discharge (n=1/3). Neurological signs including neck stiffness, abnormal posture, ataxia, facial twitching, body tremors and stupor (n=2/3). deceased (n=1/3).	Macro: Congestion, atelectatic and with abundant blood and reddish foam (n= 3/3). High viscosity mucus in larynx and serosanguinous fluid in the thorax (n= 2/3).<br><br>Micro: vascular changes in lungs including congestion, hemorrhages, intravascular coagulation and leukocytosis, probably due to drowning. Multifocal fibrinous necrotic bronchopneumonia (n= ⅓).	Macro: Meningeal congestion and multifocal hemorrhages (n= ⅓).<br>Micro: Lympho-neutrophilic meningitis (n= 3/3) Gliosis, vasculitis and hemorrhages (n=2/3) Lymphocytic plasmatic meningoencephalitis in midbrain, cerebellum and central canal. Together with multifocal neurophagia.	Macro: hæmopericardium (n= 1/3). Lymphadenomegly and multifocal discoloration of placenta. No lesions on the fetus (n= 1/3). Micro: Lympho-cytoplasmic myocarditis, probably due to stress (n= 1/3). Multifocal chorionic villus necrosis (n= 1/3).	Virus RNA detected in spinal cord (Ct= 32.5 – 34.4, n=2/3), brainstem (Ct= 27.8, n=1/3), placenta (Ct=29.3, n=1/3), fetal lung (Ct=23.9, n=1/3), fetal heart (Ct=33.2, n=1/3), fetal kidney and thymus (Ct=34.9, n=1/3).	IAV NP antigen present in brain tissue in neurons and glial cells (n= 2/3). "Present in epithelial cells of the choroid plexus, ependymal cells of ventricles and central canal of the spinal cord and in macrophages multifocally distributed in the meninges (n= 1/3). No IAV NP antigen present in heart cells (n= 1/3).<br><br>IAV NP antigen is present in trophoblast cells and maternal vessels (n= 1/3).	NA

IAV: Influenza A Virus. NA: not available. Macro: macroscopic pathological lesions. Micro: microscopic pathological lesions. PCR: polymerase chain reaction. IHC: immunohistochemistry. Ct: Cycle threshold. NP: Nucleoprotein

Table S3 continued.

Study and species	Clinical signs	Respiratory lesions	CNS lesions	Lesions in other organs	Virus localization (RT- qPCR)	Virus localization (IHC)	Transmission route
Liang et al., 2023<br><br>Harbor seal (*Phoca vitulina*), Denmark (n=1). Adult male.	Emaciated.	The heart and respiratory organs appeared unaffected.	NA	The heart appeared unaffected, whereas the abdominal organs were too decayed for thorough examination.	Virus RNA detected in the lung of the seal (Ct=28.99).	NA	NA
Stimmelmayr et al., 2024.<br><br>Polar bear (*Ursus maritimus*), United States of America (n=1).	Fair to poor body condition score. Deceased.	Lung congestion. Micro: pulmonary edema, focal lipid pneumonial.	Cerebral swelling and congestion. Micro: "granulocytic and mononuclear meningoencephalitis with microgliosis, neuronal necrosis, neuronophagia, vasculitis, and parenchymal rarefaction".	Macro: "Empty stomach. Ulcerative skin lesions (1–3-cm) ulcerative skin lesions around the left eye and oral commissure, liver congestion, moderate sanguinal pericardial and cavitary effusion". Micro: Multifocal ulcerative dermatitis.	Negative	IAV IHC: brain: cytoplasm of neurons and nuclei of microglial cells not performed on other tissues.	NA

IAV: Influenza A Virus. NA: not available. Macro: macroscopic pathological lesions. Micro: microscopic pathological lesions. PCR: polymerase chain reaction. IHC: immunohistochemistry. Ct: Cycle threshold. NP: Nucleoprotein
